# Understanding the Brain through Aging Eyes

**DOI:** 10.20900/agmr20210008

**Published:** 2021-03-01

**Authors:** Marian Blazes, Cecilia S. Lee

**Affiliations:** 1 Department of Ophthalmology, University of Washington, Seattle, WA 98104, USA; 2 Karalis Johnson Retina Center, Seattle, WA 98109, USA

**Keywords:** retina, Alzheimer’s disease, retinal biomarker, retinal imaging, dementia

## Abstract

The eye and brain share common mechanisms of aging and disease, thus the retina is an essential source of accessible information about neurodegenerative processes occurring in the brain. Advances in retinal imaging have led to the discovery of many potential biomarkers of Alzheimer’s disease, although further research is needed to validate these associations. Understanding the mechanisms of retinal disease in the context of aging will extend our knowledge of AD and may enable advancements in diagnosis, monitoring, and treatment.

The retina offers a unique opportunity to study the brain. The eye and the brain share the same embryologic origins, similar vascular systems, and immunologic functions, allowing us to evaluate what may be happening in the brain through the eye non-invasively. Retinal progenitor cells, precursors to a variety of retinal neuronal cells, originate from the neural plate during embryogenesis as do other structural and functional components of the eye [1]. The direct communication between the eye and the brain is maintained throughout life through the axons of the retinal ganglion cells, which connect with the visual cortex via the optic nerves [2]. Additionally, the retinal and brain vasculatures share similar mechanisms for protecting the neuronal cells from the circulatory system (the blood-retinal barrier and the blood-brain barrier, respectively) mediated by the retinal pigment epithelium and endothelial cells in the retinal vasculature and by endothelial cells in the cerebral vasculature [3]. The breakdown of these barriers and resulting activation of inflammatory cascades have been associated with neurodegenerative processes in both the retina and the brain such as diabetic retinopathy, age-related macular degeneration (AMD), Alzheimer’s disease (AD), Parkinson’s disease, multiple sclerosis, and stroke [4–7]. These structural and functional similarities between the retina and the brain provide potential explanations for the linked pathological processes that occur in aging eyes and aging brains.

Studying aging eyes offers a unique platform to advance our understanding of the neurodegenerative changes that occur with aging and pathologies in the brain such as AD and related dementias (ADRD). Currently, many challenges exist in AD research such as the need for histological confirmation for definitive diagnosis of AD, the expensive and invasive testing associated with biomarker-based diagnosis, and the difficulty of in vivo research on mechanisms, progression, and response to potential therapies. Thus, many researchers are looking for alternative approaches for studying AD, such as through the eye. Recent advances in retinal imaging have greatly increased our understanding of retinal structure and function in unprecedented detail. As a result, several studies have shown promising results indicating that the retina may allow for early detection of neurodegenerative changes associated with cognitive impairment and AD [8–10].

Hinton et al. were the first to demonstrate connections between neurodegeneration in the retina and the brain with histopathological evidence of ganglion cell loss and retinal nerve fiber layer (RNFL) thinning in the eyes of patients with AD [11]. Since then, many imaging modalities have been used to detect features that may distinguish AD from controls. Structural changes visible on optical coherence tomography (OCT), such as thinning of the peripapillary and macular RNFL, ganglion cell inner plexiform layer, and choroid, have been linked to mild cognitive impairment (MCI) and AD [12–14]. One of the main advantages of OCT imaging includes the ability to detect changes in retinal features providing insight into the sequence of events that occur during the development of AD. Therefore, researchers continue to determine how progression and location of RNFL thickness variations and resulting *visual* function changes, such as in contrast sensitivity, are associated with different stages of AD progression, particularly mild cognitive impairment (MCI) versus AD [15,16]. More recent studies using OCT angiography have shown that changes in the retinal and choroidal vasculature such as enlarged foveal avascular zone are associated with MCI and AD [17,18]. One study found significant choroidal thinning in mild AD while the foveal avascular zone and hemoglobin colorimetric analysis of optic nerve head perfusion did not differ from controls, suggesting that alterations in the choroidal vasculature or structure may occur earlier than the retinal changes associated with AD development [14]. Additionally, functional changes such as retinal oxygen metabolism and other metabolic alterations have been quantified and shown to be associated with AD using retinal oximetry and fluorescence lifetime imaging ophthalmoscopy [19–21]. Lastly, histopathological studies of post-mortem eyes of patients with AD demonstrated amyloid beta (Aβ) and hyperphosphorylated tau in the retina [22,23], while Aβ has also been observed in the retina of patients with AD in vivo using hyperspectral imaging. These findings highlight the possibility of evaluating potentially shared disease pathways with AD via noninvasive retinal imaging [24].

Although aging is the common risk factor, several potential mechanistic explanations exist for the relationship between neurodegenerative diseases of the eye and AD [25]. Eye disease related vision loss may contribute to cognitive decline, due to social isolation or reduced stimulation to the visual cortex, or sensory loss may increase cognitive load to the extent that overall cognition becomes impaired [26]. A common metabolic pathway related to a systemic disease such as diabetes could cause both retinal and cerebral neurodegeneration, potentially via systemic microvascular pathology or disruption of neurovascular coupling [26–28]. Simultaneous accumulation of Aβ and tau pathology in both the retina and the brain, causing localized inflammation that leads to neuronal death, is another potential mechanistic link between age-related retinal disease and AD [22]. Mitochondrial dysfunction may also play an important role in the neurodegenerative cascade seen in retinal pathologies such as AMD and diabetic retinopathy as well as in AD [29]. Both the retina and the brain are metabolically active thus highly susceptible to damage from oxidative stress caused by both aging and disease processes. Other neurodegenerative diseases such as Parkinson’s disease also demonstrate evidence of retinal mitochondrial dysfunction associated with axonal loss in the temporal optic disc containing the papillo-macular bundle, which is affected in other optic neuropathies [30]. A new imaging technique, flavoprotein fluorescence, can noninvasively measure mitochondrial dysfunction and oxidative stress in the retina, as oxidized mitochondrial flavoproteins autofluoresce in response to blue light stimulation [31,32]. Thus this type of technique may be helpful in illuminating the role of mitochondria in the AD pathology.

The activation of microglia during the inflammatory response to neuronal degeneration and protein deposition is another potential pathologic mechanism that occurs in the retina in association with AD [33]. Evidence from AD mouse models suggests that microglial activation may be an early event, and the timing of these events may be detectable on OCT as evolving changes in the thickness of specific retinal layers. Mouse models of amyotrophic lateral sclerosis have also demonstrated an increase in microglial cells surrounding retinal ganglion cells. Although microglial cells act as macrophages in response to degeneration, they are thought to induce additional neuronal damage [4,34,35]. One limitation to exploring this type of process as potential biomarkers of AD is the difficulty of obtaining in vivo retinal imaging due to factors such as media opacity (lens or cornea), participant movement (maintaining fixation during imaging is challenging for elderly adults), and concomitant age-related neurodegenerative eye disease that confound the imaging abnormalities. One potential approach for studying AD biomarkers is the development of tracer studies, in which the biomarker in question is targeted so that specific imaging of the biomarker can be obtained. Curcumin-enabled amyloid detection in the retina would be an example [26,36]. Future development of additional molecular markers that could detect cellular changes such as glial activation will be useful in obtaining further insights into pathogenesis. In addition, novel approaches such as modeling of retinal fluid dynamics associated with neurodegeneration and AD show promise as a biomarker profile that could integrate several features related to multiple pathological mechanisms [37].

Associations between eye diseases and AD that have been reported in epidemiological studies emphasize the importance of screening and studying eye pathologies in people at risk of ADRD. Both visual impairment and specific eye diseases have been associated with increased risks of AD. Several recent studies have found that the risk of dementia increased with worsening visual impairment, and impaired visual contrast sensitivity and pupillary response have been associated with AD neuropathology and risk of AD, respectively [38–42]. A review of prospectively collected, community-based cohort of older adults found that the diagnoses of AMD, diabetic retinopathy, and glaucoma were associated with higher risks of developing AD (HRs 1.46–1.67, *p*-values <0.001–0.045) than those without these eye diseases [43]. These study findings were consistent with other population-based studies linking neurodegenerative eye disease and AD [44–47], although many were limited in size or lack of well-characterized cohorts. Geriatricians and neurologists should be aware of these associations, and routine ophthalmology evaluations should be considered for older adults.

Research related to the validation of retinal biomarkers of AD has advanced rapidly but challenges still remain [48]. Many studies have been limited by small size, cross-sectional design, and exclusion of patients with concomitant eye disease, thus have produced conflicting results [16,49,50]. In addition, studies have used different cognitive assessment methods, and not all have relied on research criteria to distinguish mild cognitive impairment and/or ADRD. Similarly, standardized retinal imaging or analysis protocols do not exist yet to enable interpretation across multiple studies [16].

Moving forward, it will be essential to determine whether retinal biomarkers are specific to clinical AD, neuropathologic AD, biomarker-based AD with or without cognitive changes, or to some or all of these categories. The timing of these associations is also important; biomarkers for preclinical AD may differ from those associated with disease progression [16]. Understanding retinal vascular, structural, and metabolic alterations in relation to AD diagnosis and progression will require longitudinal studies in well-characterized cohorts [16,48]. Ultimately, large collaborations between researchers, industry partners, and funding agencies will be needed to create standardized ophthalmic imaging acquisition protocols, data collection, and data sharing mechanisms following the successes of the Alzheimer’s Disease Neuroimaging Initiative (ADNI) [51].

Much remains to be learned about the unique connection between the aging eye and the aging brain. Noninvasive retinal imaging techniques and innovative image analyses such as machine learning approaches will continue to provide new insights into the mechanisms of aging and related pathological processes. This information will extend our understanding of the brain, and vice versa. As the population continues to age, collaborations between ophthalmologists, neurologists, and gerontologists will be essential, both in patient care and in research, as the eye becomes a key resource for understanding the aging brain.

## ABBREVIATIONS

Aβ: amyloid beta
AD: Alzheimer’s disease
ADNI: Alzheimer’s disease neuroimaging initiative
ADRD: Alzheimer’s disease related dementias
AMD: age-related macular degeneration
MCI: mild cognitive impairment
OCT: optical coherence tomography
RNFL: retinal nerve fiber layer
